# Books and Magazines

**Published:** 1907-05

**Authors:** 


### Books and Magazines.

PARAFFIN IN SURGERY.—A critical and clinical study by Wm. H. Luckett, M. D., Attending Surgeon, Harlem Hospital, Surgeon to the Mt. Sinai Hospital Dispensary of New York, and Frank I. Horne, M. D., Formerly Assistant Surgeon, Mt. Sinai Hospital Dispensary. 12mo.; 38 illustrations; 118 pages. Surgery Publishing Company, 92 William street, New York City. Cloth, $2.00.

This book covers a special field in surgery of absorbing interest both to the surgeon and general practitioner. The research and original investigations made by these authors in the use of Paraffin have exploded many fallacies previously maintained. It presents the Chemistry of Paraffin, the Early Disposition of Paraffin in the Tissues, Physical State of the Paraffin Bearing on its Disposition, the Ultimate Disposition of Paraffin, Technic and Armamentarium. It thoroughly covers the use of Paraffin in cosmetic work such as Saddle, Nose Deformity, Depressed Scars, Hemiatrophia Facialis, with a large number of photographs showing cases before and after operation, with illustrations of micro-photographs of the Disposition of the Paraffin in the Tissues. It also presents other conditions of a functional character, where Paraffin can be used with service such as Incontinency of Urine, Umbilical Hernia, Umbilical and Ventral Hernia, Epigastric Hernia, Inguinal Hernia, etc. The subject is presented in a scientific yet comprehensive manner.

Full details are given as to the method of preparing the Paraffin as well as the method and manner in which it should be injected. This book presents a wide field for the use of Paraffin, and a copy should be in every physician's library. It is printed upon heavy coated book paper and attractively bound in the best quality of heavy red cloth, stamped in gold.

A PRACTICIAN'S HAND-BOOK OF MATERIA MEDICA AND THERAPEUTICS, based upon established physiologic actions and the indications in small doses. By Thomas S. Blair, M. D. Over 250 pages, bound in limp library cloth. Price, $2, net. Published by the *Medical Council,* 4105 Walnut Street, Philadelphia, Pa.

Dr. Thomas S. Blair, of Harrisburg, Pa., has written a book embodying the results of his personal study, investigation and tests in practice of the medicinal agents prepared by the pharmacists of the different schools and methods of manufacture. This is one of the most useful books to the careful, discriminating student of therapeutics that has been published in many a year. No adequate idea of a book can be obtained without a thorough reading of it.

---

MODERN MEDICINE.—Its Theory and Practice. In Original Contributions by American and Foreign Authors. Edited by William Osler, M. D., Regius Professor of Medicine in Oxford University, England; formerly Professor of Medicine in Johns Hopkins University, Baltimore; in the University of Pennsylvania, Philadelphia, and in McGill University, Montreal. Assisted by Thomas McCrea, M. D., Associate Professor of Medicine and Clinical Therapeutics in Johns Hopkins University, Baltimore. In seven octavo volumes of about 1000 pages each; illustrated. Volume I just ready. Price, per volume, cloth, $6, net; leather, $7, net; half Morocco, $7.50, net. Lea Brothers & Co., Publishers, Philadelphia and New York, 1907-1908.

From every viewpoint, scientific, literary and practical, this new work just coming from the press promises to be the most important ever undertaken in medicine. This statement, though broad, is not without justification. The various *systems* of the past, by gathering and disseminating the best knowledge of their day, powerfully stimulated medical thought, expanding it in directions hitherto scarcely known, roundng it out here, correcting there, revolutionizing elsewhere. A complete restatement has become necessary to convey a grasp of the present development of medical science and art. Fortunately, this is now about to be done, and under the best auspices. Professor Osler combines every quality essential for leadership in such an enterprise. Circular in the sweep of his knowledge, intuitive in his perception of the place and bearing of each detail, he is abreast of every advance. Familiar with the literature of medicine, he is also acquainted with the personnel of the leaders in the various lines of investigation. So recognized, he has been able to unite them in a skillfully planned work covering the whole domain. He has never lost sight of the proper objective. In his introduction he writes, "It is designed primarily for the practitioner who wishes to keep himself informed of the existing state of our knowledge in clinical medicine. The first consideration in a work of this kind is that it shall be helpful. * * * The authors will be found to have simplified abstruse and complicated knowledge, and to have presented it in a form readily assimilable by the men who have to use it. The hope of the profession is with the men who do its daily work in practice. Our labors are in vain —all the manifold contributions of science, the incessant researches into the complex problems of life, normal and perverted, the profound and far-reaching conclusions of the thinkers and originators —all these are sounding brass and tinkling cymbals unless they result in making men better able to fight the battle against disease, better equipped for their ministry of healing. The carrying out of new methods of treatment, the exchange of the old for the new weapons and methods of warfare, these rest with the rank and file of the profession who make effective and translate into practice the new knowledge." No strain of nihilism breathes in these words, but rather the spirit of the enthusiastic seer and leader. Imbued with the purpose of giving all his powers to this effort for the benefit of his race, Dr. Osler has inspired his collaborators with equal zeal, and the result has surpassed even his expectations. The first volume is now before the profession for practical use, the second will be ready in June, and the succeeding volumes will appear at intervals of three months.

The first volume, which has now come to hand, contains an introduction by the editor himself, which he entitles "The Evolution of Internal Medicine"—a most interesting history of medicine from pre-Hippocratic times to the present day, tracing the growth of the science and art in the various schools and countries which have contributed to build up the existing structure of knowledge. To this is added a forecast of the lines on which further development would most fruitfully proceed. The whole introduction is, of course, scholarly and epigrammatic, marked by the keen observation of the trained physician who is also a man of the world, full of wisdom and altogether delightful reading.

Dr. J. G. Adami, of Montreal, begins the body of the volume with an article on "Heredity and Predisposition," in which he asserts, with much emphasis: "It is impossible for there to be inheritance proper of infectious diseases. There is no such thing as inherited smallpox, inherited tuberculosis, or hereditary syphilis. * * * This, however, is not the same as stating that no inheritance of any order can occur in connection with specific disease." The article is thoroughly up to date, and from the charm of the author's style, this most important subject is made very easily understood.

"Auto-intoxications," from the pen of Dr. Alonzo Englebert Taylor, of San Francisco, is another of these conclusive and exhaustive articles on a difficult subject which will make Osler's Modern Medicine the recognized authority for the next quarter of a century. His treatment of the question of Gastro-Intestinal Auto-Intoxication is of the most illuminating character, and is full of helpful suggestions to the general practitioner in the treatment of diseases consequent on malnutrition.

Nearly sixty pages are devoted by Dr. Charles F. Craig, U. S. A., to the consideration of "Malarial Fevers." The article is comprehensive, accepting, of course, the theory of transmission by mosquitoes, and pointing out that a fever which is not cured by the proper administration of quinine is not of malarial origin. His suggestions as to prophylaxis are in every respect valuable.

Other thoroughly practical clinical articles are those by Dr. Thomas B. Futcher, of Johns Hopkins, on "Diabetes and Gout"; by Dr. J. M. Anders on "Obesity"; and by Dr. George F. Still, of London, on "Rickets." The scientific physician will regard the profoundly scholarly article on "Metabolism, Normal and in Disease," by Chittenden, of Yale, as of fundamental value. There are excellent articles, also, by Drs. Alfred Gordon and David L. Edsall, of Philadelphia; Alexander Lambert, of New York; F. G. Novy, of Ann Arbor; James H. Wright, of Boston, and others.

Modern Medicine, the best fruitage of the new era, is a work for every physician who would keep qualified for the full discharge of the duties attaching to the most responsible of all professions.

---

PHYSICAL DIAGNOSIS—With Case Examples of the Inductive Method. By Howard S. Anders, M. D.. With 88 illustrations in the text and 32 plates. Cloth; 450 pages. Price not stated. D. Appleton & Co., New York and London.

This is an important subject and needs a bold and masterful handling. We believe that heretofore there has not been a really good book upon this subject. This work will clear the way and act as a guide for the student and the practitioner who must perfect themselves in this important branch of medical science.

Proper emphasis has been given by Dr. Anders to the value of inspection, a point too often neglected, and also mensuration, especially as is useful to medical examiners for life insurance. Methods of percussion are described in detail. There is a most valuable article on stethoscopes and the relative advantages and disadvantages in auscultation. There are other tables of differential physical diagnosis, and a graphic chapter on heart murmurs. There are many plates of X-ray illustrations, the finest ever shown in a work upon physical diagnosis.

We have not space to mention all of the excellent points in this work, but we believe it will take a place as the standard authority upon the subject.

